# Second-Look Surgery for Intestinal Salvage in a Neonate With Midgut Volvulus and Intestinal Malrotation: A Case Report

**DOI:** 10.7759/cureus.88600

**Published:** 2025-07-23

**Authors:** Oscar Alvarez-Lopez, Korel Hernandez-Lopez, Jorge A Castañeda, Eila N Herrera

**Affiliations:** 1 General Surgery, Hospital General de Occidente, Zapopan, MEX; 2 General Surgery, Hospital Angeles del Carmen, Guadalajara, MEX

**Keywords:** congenital intestinal malrotation, intestinal malrotation, midgut volvulus, second-look surgery, small bowel obs, small bowel obstruction

## Abstract

Intestinal malrotation is described as an abnormal positioning of intestinal loops within the peritoneal cavity, caused by defective rotation around the superior mesenteric artery. This results in a short mesenteric root, which predisposes to midgut volvulus when the intestine twists on its axis. We present a case of a five-day-old female with bilious vomiting, abdominal distension, and radiographic signs of obstruction. Surgical intervention was decided, revealing midgut volvulus associated with intestinal malrotation and questionable intestinal viability. A second-look surgery was scheduled at 48 hours, during which the Ladd procedure was completed without requiring intestinal resection. In conclusion, we emphasize the importance of early surgical intervention and the use of techniques that may prevent irreversible intestinal necrosis and the need for extensive resections.

## Introduction

Currently, the incidence of developmental intestinal anomalies resulting from interrupted embryologic rotation is not well known, as patients may remain asymptomatic [1,2]. The spectrum of intestinal rotation abnormalities arises from disruptions in the sequence of herniation, rotation, and fixation of the midgut. Malrotations occur in approximately 0.2-1% of the population [3]. Classical malrotation is characterized by the duodenojejunal junction being located in the right upper quadrant and the cecum in the upper abdomen, fixed by Ladd’s bands. This leads to a narrowed mesenteric base, which predisposes to midgut volvulus. The incidence of malrotation with volvulus decreases with age. A total of 85.7% of neonates had midgut volvulus at the time of presentation [1,3-5]. This volvulus occurs around the mesenteric pedicle, causing torsion of the superior mesenteric artery and vein, representing a critical condition that may lead to ischemia of a large segment of intestine, potentially requiring extensive resections with fatal consequences [1]. Therefore, a diagnostic suspicion is essential for the timely recognition and surgical treatment, which is crucial for reducing morbidity and mortality associated with this pathology.

## Case presentation

**Figure 1:**
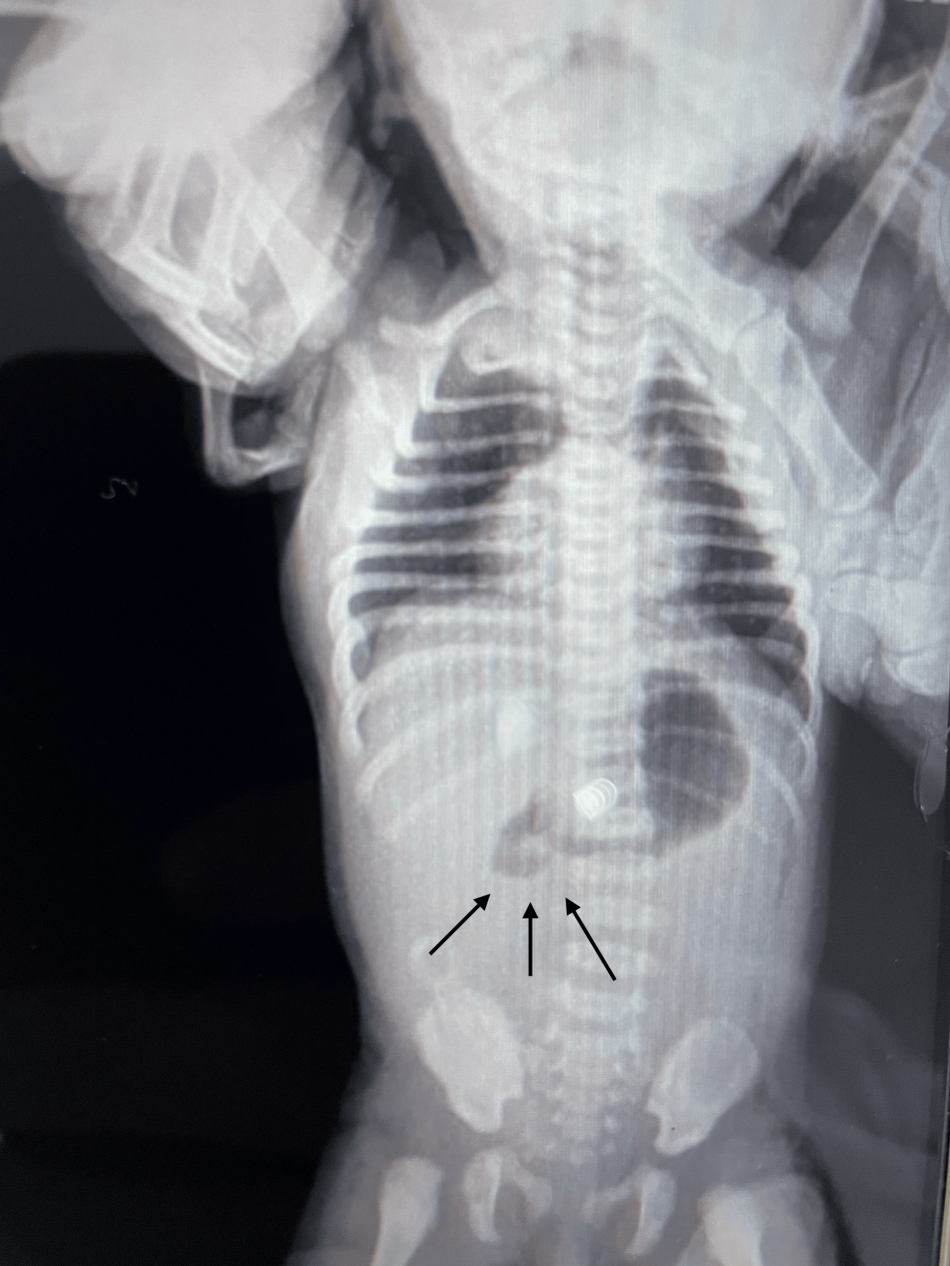
Abdominal X-ray showing absence of distal bowel gas (arrows), suggestive of intestinal obstruction.

A five-day-old female, weighing 5.3 kg, and born to a 25-year-old woman without personal history of diseases, presented to the emergency department with bilious vomiting, abdominal distension, and absence of bowel movements for 48 hours. Vital signs were within normal limits for age. Laboratory studies revealed leukocytosis (14,000/μL); abdominal radiography showed an absence of distal gas, suggestive of intestinal obstruction. No signs of peritoneal irritation were present. An exploratory laparotomy was performed; prior informed consent was obtained, revealing intestinal loops twisted around their mesentery, consistent with midgut volvulus (Figure 1).

**Figure 2:**
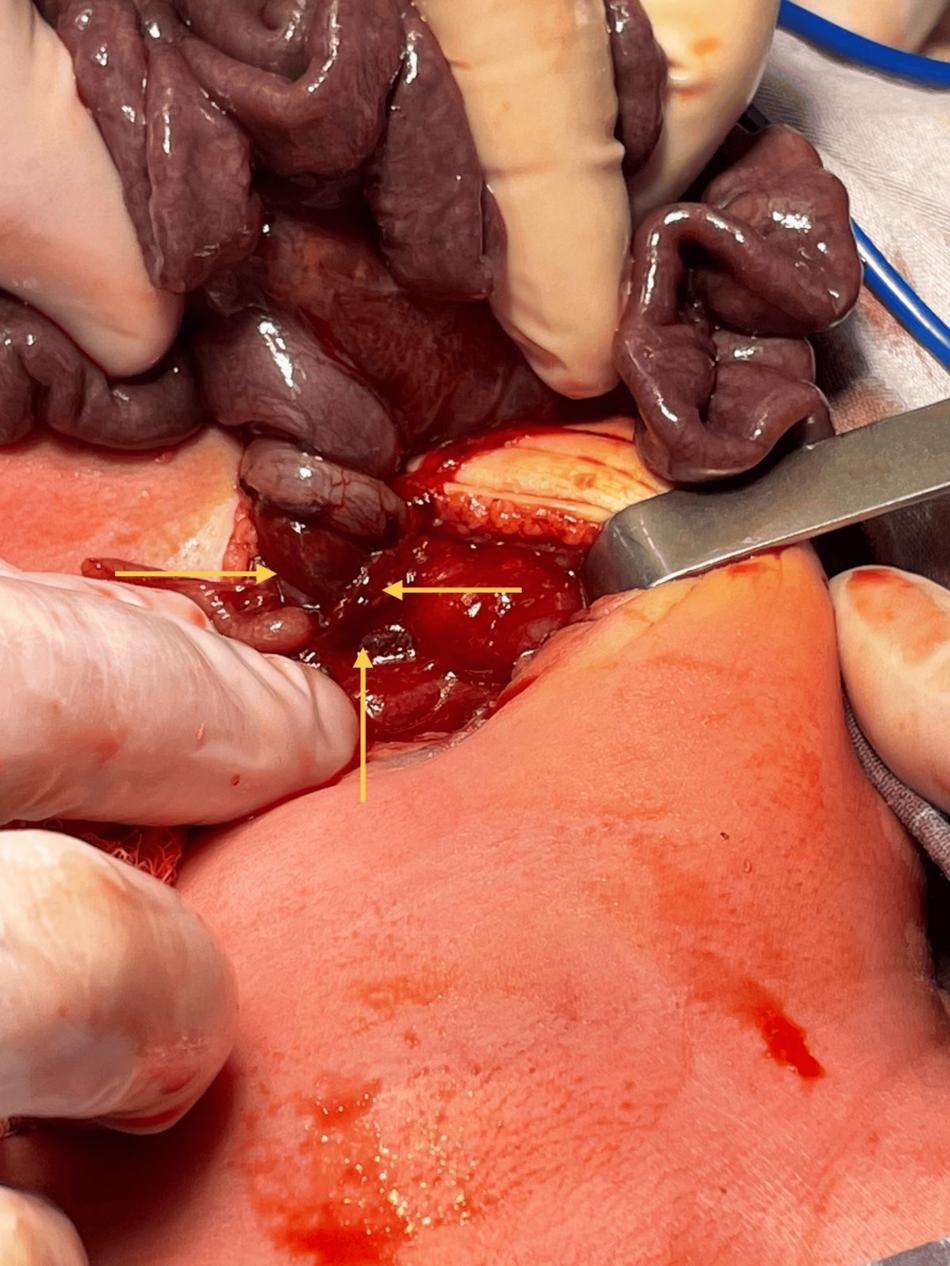
Torsion of the small intestine and its mesentery around the superior mesenteric artery (arrows), consistent with midgut volvulus. A torsion of the small intestine and its mesentery around the superior mesenteric artery was observed, secondary to a narrowed mesenteric base. This was consistent with midgut volvulus, which is associated with vascular occlusion and secondary intestinal ischemia.

**Figure 3:**
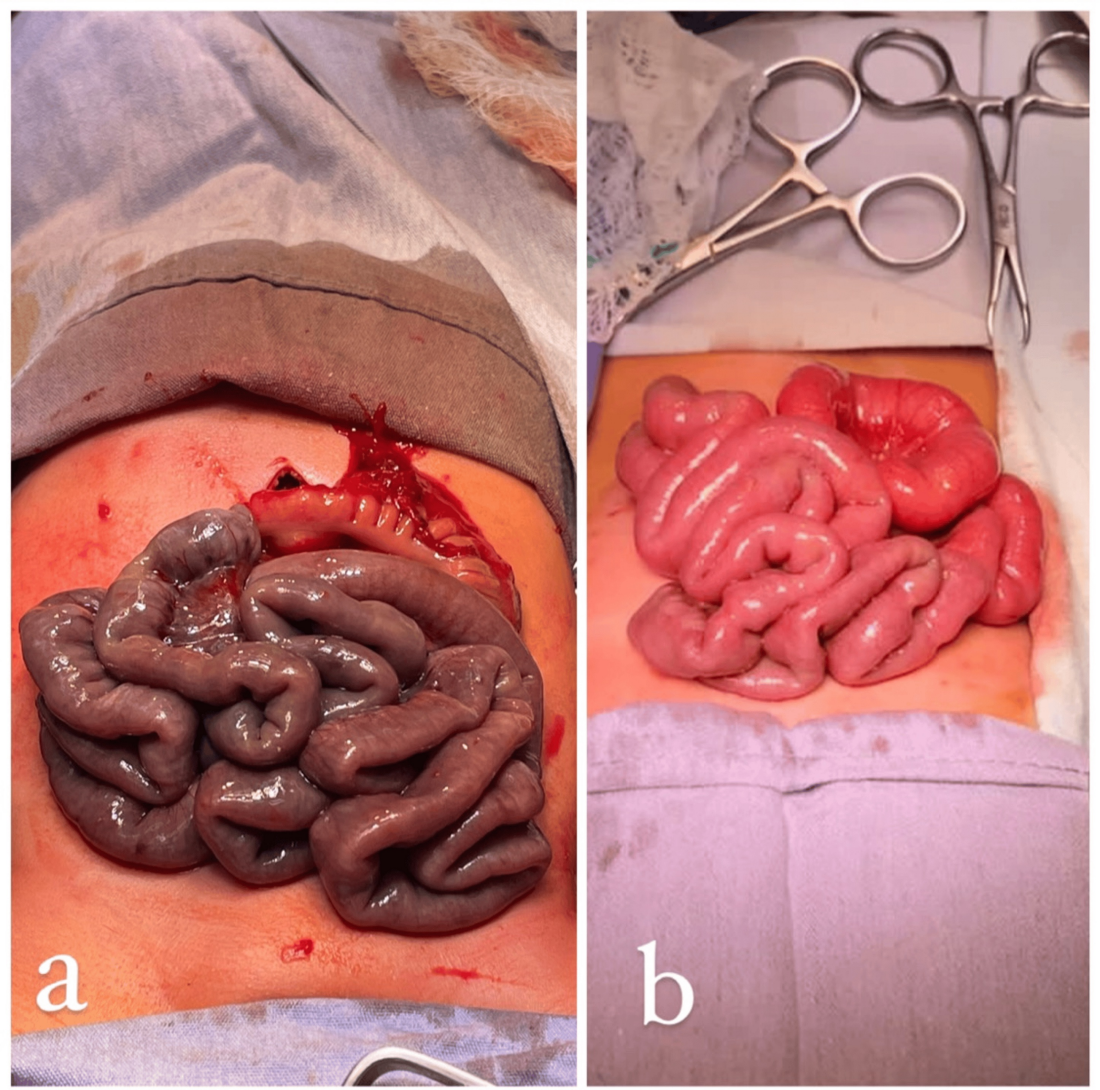
Comparison of intraoperative intestinal loop viability. (a) Findings during the first laparotomy reveal small bowel loops with ischemic changes, as well as a cecum positioned in the right upper quadrant, consistent with intestinal malrotation. (b) Findings during the second-look laparotomy show small bowel loops with a healthy pink coloration, indicating restored viability.

No signs of peritoneal irritation were present. An exploratory laparotomy was performed, revealing intestinal loops twisted around their mesentery, consistent with midgut volvulus (Figure 2). Upon detorsion, ischemic changes were noted throughout the intestinal loops. The cecum was located in the left upper quadrant and fixed to the abdominal wall by Ladd’s bands, which were divided. The mesentery was broadened, but ischemic signs persisted. A second-look procedure was scheduled at 48 hours to complete the Ladd procedure with appendectomy and reassess intestinal viability. Full recovery of the intestine was confirmed, avoiding the need for resection (Figures 3a, 3b).

The patient had an uneventful postoperative course, with oral intake resumed on postoperative day six and discharge on day seven. Follow-up for seven months revealed no complications.

## Discussion

Currently, midgut volvulus is associated with high morbidity and presents with variable clinical signs. The most common is bilious vomiting, typically appearing suddenly between the third and fifth day of life. It should be considered a surgical emergency until proven otherwise [6,7], as it may lead to intestinal necrosis, perforation, peritonitis, and other symptoms, which can be fatal, making timely treatment essential [1,7]. The most sensitive diagnostic test remains contrast radiography showing a “corkscrew” pattern [5]. The “whirlpool sign” observed on CT or Doppler ultrasound, representing clockwise twisting of the small intestine and mesentery around the superior mesenteric artery, is considered pathognomonic but is present in only 64% of cases [5,7]. When midgut volvulus is suspected, urgent surgical exploration should be performed [1,7,8], even without radiographic confirmation [1], as it can prevent massive resections and serious complications, such as intestinal failure or short bowel syndrome [2].

In this case, initial assessment with plain abdominal radiography allowed early diagnosis of intestinal obstruction with suspected vascular involvement, prompting surgical exploration. The treatment of choice remains the Ladd procedure [1,4,7,9,10]. In cases of suspected volvulus, open surgery is preferred [9]. The surgical principles include counterclockwise detorsion in the presence of volvulus, division of Ladd’s bands if present, broadening of the mesenteric root, and repositioning of the small intestine to the right quadrants and the colon to the left [1,9]. Appendectomy is usually performed. Intestinal viability should be assessed both after detorsion and at the end of the procedure [1].

In acute volvulus, the intestine often presents with congestion and hyperemia, giving it an ischemic appearance. After detorsion and a period of observation, vascularity often recovers, and the extent of resection may be less than initially estimated [10]. In cases where intestinal viability is questionable, as in our patient, second-look laparotomy has been successfully reported as a method to salvage varying lengths of small intestine [10-12]. This technique is more commonly used in cases of necrotizing enterocolitis [11] and is rarely used in volvulus cases [10,12,13]. In this case, ischemic changes persisted in extensive intestinal segments after detorsion, so a second-look exploratory laparotomy at 48 hours was performed, with complete intestinal recovery. Although rare, similar cases have been reported, such as by Jan et al., which demonstrate that additional segments of small intestine can be preserved using this technique [10]. This is vital to avoid complications, such as short bowel syndrome, a leading cause of morbidity and mortality in these patients.

## Conclusions

The epidemiology of volvulus associated with intestinal malrotation is poorly understood. In suspected cases, early surgical intervention is essential. Techniques, such as second-look laparotomy, should be considered, as they may prevent irreversible intestinal necrosis and the need for extensive resections. Despite its application in mainly isolated cases, routine use should be considered when ischemic segments with doubtful viability are encountered.
